# Development of a mobile application for vancomycin dosing calculation: A useful tool for the rational use of antimicrobials

**DOI:** 10.1016/j.rcsop.2022.100115

**Published:** 2022-02-05

**Authors:** Tácio de Mendonça Lima, Millena Padela da Silva, Luan Donato Silva Luz, Thais Cristina Amorim Estevão Soares, Etielle Silvestre Dantas, Gabriela Felix Teixeira, Rafael Henrique de Souza Costa, Sérgio Henrique Monte Santo Andrade

**Affiliations:** a Department of Pharmaceutical Sciences, Federal Rural University of Rio de Janeiro, Seropédica, RJ, Brazil; b School of Pharmacy, Federal Rural University of Rio de Janeiro, Seropédica, RJ, Brazil; c Graduate Program in Administration and Management of Pharmaceutical Service, Fluminense Federal University, Niteroi, RJ, Brazil; d Faculty of Estacio, Belem, PA, Brazil; e Salt Engineering and Automation LDTA, Tv. Rui Barbosa 571, sl. 3, Belem, Para, Brazil

**Keywords:** Mobile applications, Vancomycin, Drug dosage calculation, Methicillin-resistant *Staphylococcus aureus*

## Abstract

**Background:**

Mobile applications (app) provide many benefits for healthcare professionals, making them a useful support clinical decision system.

**Objectives:**

To describe the development of a mobile app, CalcVAN, to calculate vancomycin dosage regimens for adult and pediatric patients.

**Methods:**

This study is a technological production research to develop a mobile app through the rapid prototyping type for the Android system in the Brazilian context. The mobile app structure was developed in four steps: 1) conception, including the needs assessment, the target audience, the literature search, and the definition of contents; 2) prototype planning, including the definition of topics and writing of modules, the selection of media, and the layout; 3) production of the mobile app, including the selection of multimedia tools, the navigation structure, and planning of environment configuration; and 4) make the mobile app available.

**Results:**

The CalcVAN has six screens, containing the vancomycin dosing calculator for adult and pediatric patients based on weight and estimated creatinine clearance parameters. Moreover, the mobile app is free and can be used without internet connection.

**Conclusions:**

A free mobile app was developed to calculate vancomycin dosage regimens for inpatients. This tool assists to optimize the vancomycin dosing, contributing to the antimicrobial stewardship.

## 1 Introduction

*S. aureus* (*S. aureus*) is an important pathogen that may cause serious infections in inpatients, including osteomyelitis, endocarditis and bacteremia, due multi-drug resistant strains, such as Methicillin-Resistant *S. aureus* (MRSA).^1^ Among the available options for treatment of these infections, vancomycin remains the drug of choice for many cases, being less expensive than other alternatives, such as linezolid, daptomycin and tigecycline.^2^ However, vancomycin has a narrow therapeutic index, which is defined as a small difference between the therapeutic and toxic doses. Therefore, individualization of drug dosage regimens must be performed based on clinical characteristics of each patient, such as weight and estimated creatinine clearance.^3^

In this sense, the best knowledge of the pharmacokinetics and pharmacodynamics (PK/PD) of vancomycin allowed new proposals for optimizing the dose.^4^ The AUC_24h_/MIC (area under the curve in 24 h to the minimum inhibitory concentration) ratio is considered the best PK/PD index to determine vancomycin effectiveness, with a value of ≥400 being desirable.^5^^,^^6^ Nonetheless, the vancomycin trough serum concentration of 15 to 20 mg/L was adopted due to the complexity of calculating the AUC_24h_/MIC ratio in clinical practice.^6^ Thus, some studies suggested new dosage regimens, including nomograms, to increase the probability of achieve the desirable therapeutic range as well as contribute to the antimicrobial stewardship.^2^, ^3^, ^4^

In the global context, mobile technology presents an opportunity to assist healthcare professionals in health care.^7^ The rapid growth of mobile health applications (mHealth app) demonstrates that developers see a current market for mobile health, offering benefits such as practicality.^8^ On other hand, the mHealth apps available in app stores has increased rapidly in recent years, making it difficult to choose reliable mobile apps.^9^

Regarding the antimicrobial stewardship, it is known that the use of mobile apps to assist the education and training of health professionals in relation to the prescribing of antimicrobial is being used more and more.^10^ A mobile app to calculate drug dosage regimens with quality and reliable information becomes an attractive tool for healthcare providers. There are a good number of web sites on vancomycin dosing calculation. However, the use of these tools through smartphones does not seem to be intuitive, very limited with calculus configurations, in addition to requiring internet for full use. There are few mobile apps in the app stores, most of which are paid or require some kind of subscription. Finally, no mobile apps were developed in the Portuguese language. Therefore, the aim of this study was to describe the development of a mobile app to calculate vancomycin dosage regimens for patients with severe MRSA infections in a Brazilian context.

## 2 Method

### 2.1 Study design and working team

A technological production through the rapid prototyping type was carried out to develop a mobile app to calculate vancomycin dosage regimens for inpatients.

The working team was comprised of two areas: the content experts, including one university professor in pharmaceutical sciences, one clinical pharmacist, and four pharmacy students; and the software experts, including one university professor in computer and electrical engineering and one app developer.

### 2.2 Phases of development

The mobile app development was performed according to the agile method described by Dingsøyr et al.,^11^ following four well-defined steps: 1) conception, 2) prototype planning, 3) mobile app development, and 4) availability of the mobile app.

#### 2.2.1 Conception

The first step included including the needs assessment, the target audience, the literature search, the definition of content, and the technological infrastructure analysis.^11^ Thus, a literature search was conducted to identify relevant studies published between January 2009 and September 2021 in the MEDLINE (PubMed), Scopus, and LILACS (Latin American and Caribbean Health Sciences Literature) databases, with combinations of terms relating to vancomycin dosing, treatment, and guidelines. The data records were screened and selected by one author. In addition, consolidated guidelines and other databases were used. Moreover, a search was carried out to identify similar free mobile apps in the two main app stores: Google Play (Android) e Apple Store (iOS).

The target audience was defined as the healthcare professionals who provide care to patients with severe MRSA infections.

The definition of content was performed through virtual working team meetings. The content experts presented a diagram with relevant information (mobile app name, concepts, nomograms, and references), using an online software (https://www.lucidchart.com/), in order to guide the building of the mobile app. The mobile app was called CalcVAN.

Based on all of the information presented by content experts, the app developer identified the necessary technological infrastructure to develop the mobile app, such as graphical user interface (GUI), cloud data storage, internet, and application monitoring.

#### 2.2.2 Prototype planning

The second step consisted the defining topics, writing modules and the design of the interface (layout).^11^ The design basis of the mobile app, including the layout of all screens with information about vancomycin dosing calculation, was planned by content experts through virtual meetings. The official language chosen was Portuguese. A presentation in PowerPoint was performed to improve more understanding of the app developer.

#### 2.2.3 Mobile app development

The third step included the selection of apps tools, definition of the navigation structure, and planning of environment configuration.^11^ The interface development tools of the mobile app were created by the app developer after the information provided by content experts. For this purpose, the app developer used a layered application architecture model, specifically presentation layer, so that users can access its services and functionality. The mobile app was initially developed for Android system, using Java language. Software Development Kit (SDK) for Android devices, such the Integrated Development Environment (IDE) of the Android Studio, was also used in the development of the mobile app.

#### 2.2.4 Availability of the mobile app

The last step consisted in the configuration of technological tools and resources, as well as the construction of an environment for downloading the mobile app and make it available by a target audience.^11^ The configuration of technological tools and resources process were conducted by all working team, with monthly feedback by the app developer, until beta version was finalized. The mobile app was registered within the Brazilian National Institute of Industrial Property (INPI), with the protocol number BR: 29409191942133660, and it is available online at the website (https://sites.google.com/view/calcvan/).

## 3 Results

**Fig. 1:**
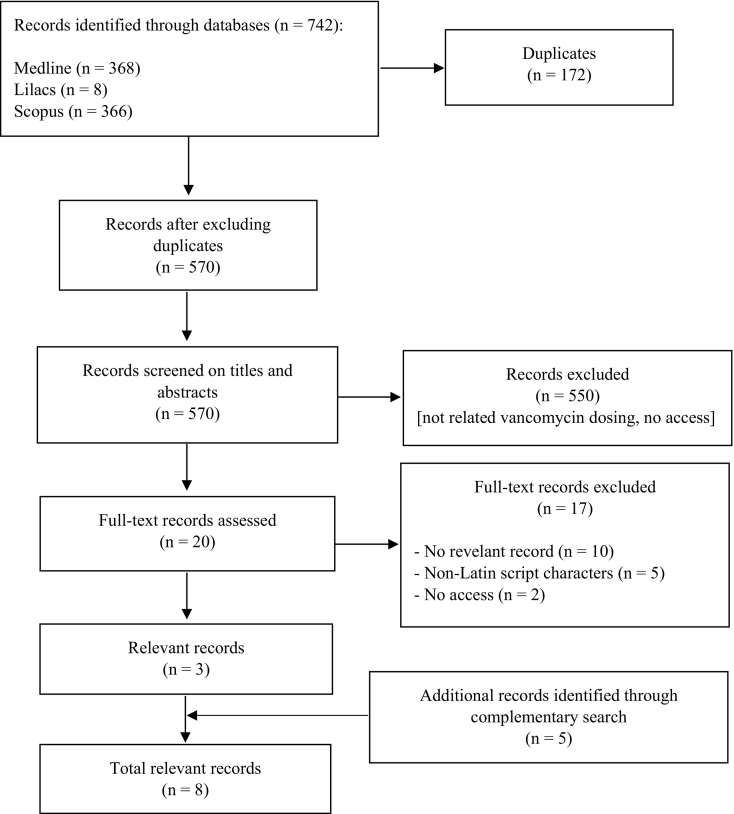
Flowchart of the relevant records to integrate the theoretical framework of the mobile app.

The literature search identified relevant studies to integrate the theoretical framework of the mobile app. A flowchart of the literature search is shown in Fig. 1. In summary, one recent consensus guidelines on therapeutic monitoring of vancomycin,^6^ two hospital protocols on vancomycin use,^12^^,^^13^ two studies on vancomycin dosing nomograms^4^^,^^14^ and one review on use of vancomycin nomograms in different populations,^5^ as well as consolidated databases, such as Sanford^15^ and Micromedex,^16^ were used in the mobile app content. In addition, two free mobile apps (Vancomycin Dose Calculator for apple device and Vancomycin Solutions for android device) were found in Brazilian app stores.

The CalcVAN app was developed for devices operating by Android system. The mobile app has six screens, in Portuguese language, containing the vancomycin dosing calculator for adult and pediatric patients as well as information of the working team and references of the literature. The healthcare professional must input values for the actual weight and estimated creatinine clearance for automatic display of the vancomycin dosage regimen. It is important to note that the mobile app was designed to calculate dosage regimens for patient with severe MRSA infections, excluding other conditions such as *Clostridium difficile* diarrhea, infection of skin and/or subcutaneous tissue, and surgical prophylaxis. Table 1, Table 2 present the vancomycin nomograms used to calculate the doses.

**Table 1:** Vancomycin dosing nomogram for adult patients according to total body weight and renal function.

		Maintenance dose										
		Estimated creatinine clearance (mL/min)										
Weight (kg)	Loading dose	< 10	10–19	20–29	30–39	40–49	50–59	60–69	70–79	80–99	100–199	≥ 120
50–60	1500 mg	1000 mg	500 mg	750 mg	500 mg	500 mg	750 mg	750 mg	1000 mg	1250 mg	1750 mg	1750 mg
		q96h	q24h	q24h	q12h	q12h	q12h	q12h	q12h	q12h	q12h	q12h
61–70	1500 mg	1250 mg	500 mg	750 mg	500 mg	750 mg	750 mg	1000 mg	1250 mg	1000 mg	1750 mg	1250 mg
		q96h	q24h	q24h	q12h	q12h	q12h	q12h	q12h	q8h	q12h	q8h
71–80	2000 mg	1250 mg	500 mg	1000 mg	750 mg	750 mg	1000 mg	1000 mg	1500 mg	1000 mg	1250 mg	1000 mg
		q96h	q24h	q24h	q12h	q12h	q12h	q12h	q12h	q8h	q8h	q6h
81–90	2000 mg	1500 mg	750 mg	1250 mg	750 mg	1000 mg	1000 mg	1250 mg	1500 mg	1750 mg	1000 mg	1000 mg
		q96h	q24h	q24h	q12h	q12h	q12h	q12h	q12h	q12h	q6h	q6h
91–100	2250 mg	1750 mg	750 mg	1250 mg	750 mg	1000 mg	1250 mg	1500 mg	1500 mg	1000 mg	1000 mg	1000 mg
		q96h	q24h	q24h	q12h	q12h	q12h	q12h	q12h	q6h	q6h	q6h

The vancomycin infusion rate was applied according to doses: 60 min of infusion for doses ≤1.0 g, 90 min for 1.1 to 1.5 g, 120 min for 1.6 to 2.0 g, and for doses >2.0 g the infusion rate was around 1.0 g/h.

Reference: Lima et al. 2014.^4^

**Table 2:** Vancomycin dosing nomogram for pediatric patients according to total body weight and renal function.

			Maintenance dose			
			Estimated creatinine clearance (mL/min)			
Weight (kg)	Loading dose	< 15	15–29	30–59	60–89	≥ 90
2.5–4.5	100 mg	25 mg q168	25 mg q8 or 12 h	25 mg q8 or 12 h	50 mg q8 or 12 h	50 mg q8 or 12 h
4.6–12.0	250 mg	100 mg q168	150 mg q24	150 mg q12	150 mg q8	150 mg q6
12.1–18.0	500 mg	150 mg q168	250 mg q24	250 mg q12	250 mg q8	250 mg q6
18.1–30.0	750 mg	250 mg q168	250 mg q24	250 mg q12	250 mg q8	500 mg q6
30.1–40.0	1000 mg	500 mg q168	500 mg q24	500 mg q12	500 mg q8	500 mg q6
40.1–50.0	1250 mg	500 mg q168	750 mg q24	750 mg q12	750 mg q8	750 mg q6
50.1–60.0	1500 mg	500 mg q168	750 mg q24	750 mg q12	750 mg q8	750 mg q6
60.1–70.0	2000 mg	500 mg q168	1000 mg q24	1000 mg q12	750 mg q8	750 mg q6

* Consider q8 for patients with postnatal age > 7 days and q12 for patients with postnatal age < 7 days. The vancomycin infusion rate was applied according to doses: 60 min of infusion for doses ≤1.0 g, 90 min for 1.1 to 1.5 g, 120 min for 1.6 to 2.0 g.

Reference: Silva et al., 2021.^14^

**Fig. 2:**
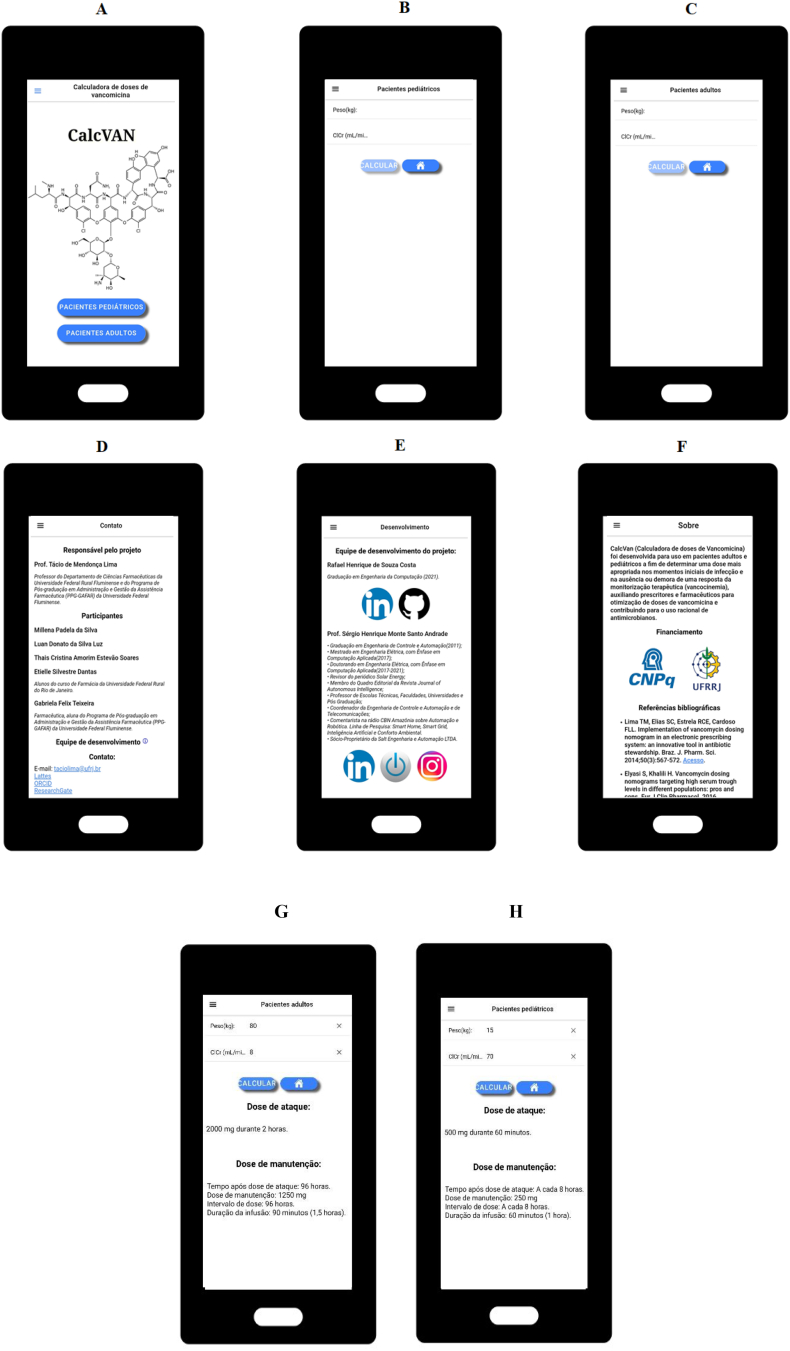
Screenshots of the CalcVAN app. A. Main screen. Subtile: *Calculadora de doses de vancomicina* is Vancomycin dosing calculator, *Pacientes pediátricos* is Pediatric patients, *Pacientes adultos* is Adult Patients. B. Vancomycin dosing calculator for adult patients. Subtitle: *Pacientes adultos* is Adult Patients, *Peso* is Weight, *ClCr* is Estimated creatinine clearance. C. Vancomycin dosing calculator for pediatric patients. Subtitle: *Pacientes pediátricos* is Pediatric patients, *Peso* is Weight, *ClCr* is Estimated creatinine clearance. D. Contact support. Subtitle: *Contato* is Contact, *Responsável pelo projeto* is Responsible by the project, *Participantes* is Participants. E. Mobile application developer team. Subtitle: *Equipe de desenvolvimento do projeto* is Mobile application developer team. F. About the CalcVAN app. Subtitle: *Sobre* is About, *Financiamento* is Source of Funding, *Referências bibliográficas* is References of the literature. G. Example of the vancomycin dosing calculator of the CalcVAN app in adult patient. Subtile: *Pacientes adultos* is Adult Patients, *Peso* is Weight, *ClCr* is Estimated creatinine clearance, *Dose de ataque* is Loading dose, *Durante 2 horas* is During 2 h, *Dose de manutenção* is Maintenance Dose, *Tempo após dose de ataque* is Time after loading dose, *Intervalo de dose* is Dosing interval, *Duração da infusão* is Rate of infusion, *Horas* is hours, *Minutos* is Minutes. H. Example of the vancomycin dosing calculator of the CalcVAN app in pediatric patient. Subtile: *Pacientes pediátricos* is Pediatric Patients, *Peso* is Weight, *ClCr* is Estimated creatinine clearance, *Dose de ataque* is Loading dose, *Durante 60* min*utos* is During 60 min, *Dose de manutenção* is Maintenance Dose, *Tempo após dose de ataque* is Time after loading dose, *Intervalo de dose* is Dosing interval, *Duração da infusão* is Rate of infusion, *Horas* is hours, *Minutos* is Minutes.

The users can directly access the vancomycin dosing calculators through the main screen. Moreover, the CalcVAN app features a side menu that facilitates navigation of the users. Finally, the mobile app was developed to be free, to be used without internet connection and to use 29 Megabytes of device storage. The screens of the CalcVAN app are shown in Fig. 2.

## 4 Discussion

To the best of our knowledge, this is the first free mobile app developed to calculate vancomycin dosage regimens for adults and pediatrics patients with severe MRSA infections. The CalcVAN app has six screens, without the need for the internet connection, containing two simple calculators. The tool assists physicians and pharmacists to optimize the dosage calculation and determine the most appropriate doses, especially in early stages of the MRSA infections, and in the absence or delay of the vancomycin therapeutic monitoring.

In fact, the use of smartphones is a worldwide trend for all people and the health professionals would not be left out. These devices offer plenty of mobile apps, including for health.^17^ In this context, some studies showed their use for antimicrobial stewardship. Hoff et al.^18^ reported the implementation of a mobile clinical decision support (CDS) app to augment local antimicrobial stewardship. Ketcherside et al.^19^ assessed feasibility and acceptance of a cloud-based mobile app for antimicrobial stewardship and infection control. Schönherr et al.^20^ assessed the additional benefits of the mobile apps in the impact of institution-specific guidelines for antimicrobials on doctors' prescribing. In this study, the development of a mobile app to calculate vancomycin dosing contributes for the antimicrobial stewardship programs, since the optimization of dosage regimens is one of the strategies for these programs.^21^

Moreover, we found two similar free mobile apps (Vancomycin Dose Calculator and Vancomycin Solutions) for vancomycin dosage calculator in the app stores. However, CalcVAN has advantages over them, since it has vancomycin dosage calculator for pediatric patients, it calculates dosage regimens for patients on dialysis or severe renal disease conditions, and uses only two parameters (actual weight and estimated creatinine clearance).

The content embedded in the mobile app is crucial for its credibility. A review of Rodrigues et al.^7^ reported that few hepatitis C apps describe the use of guidelines and/or protocols, similar to the findings of the Bicalho and Lima^22^ with HIV apps. In this study, the mobile app was developed following four steps, including the comprehensive literature search. The use of up-to-date and trustworthy sources ensure the evidence-based information and safety of the user. In addition, the layout of mobile app was clearly and objectively designed, providing a greater user engagement.^23^

One advantage of the CalcVAN app is that it is digital and in a small size. This aspect is important because takes up less the storage space, improving the mobile phone agility.^24^ Moreover, offline operation is another positive aspect, primarily in developing countries, such as Brazil, since users who encounter internet connection problems can still use the app.^7^ Finally, the mobile app was developed by a working team consisting of pharmaceutical, engineers and app developer experts. Interaction among specialists contribute to the mobile app content as well as explore methods to improve the user adherence and engagement.^7^^,^^22^^,^^25^

This study has some limitations. First, this app was developed for a Brazilian context in spite of the theoretical framework used for the app content is accepted in worldwide. Second, the Portuguese language used in the mobile app restricts the use for native Portuguese users. Third, the CalcVAN was only developed for Android system, even though that this operating system is compatible with most mobile devices, which makes the app accessible to more users. Finally, the mobile app usability testing was not performed.

## 5 Conclusions

A free mobile app was developed to calculate vancomycin dosage regimens for inpatients. This tool has great potential to be used in clinical practice by health professionals, such physician and pharmacist, once assists to optimize the vancomycin dosing, contributing to the antimicrobial stewardship. As perspective, the CalcVAN will be validate through of the mobile app usability testing by the users, will update your version adding new languages, such English and Spanish, and make it available in the Google Play Store. Moreover, future research is needed to confirm whether the performance of healthcare providers is improved and inappropriate vancomycin prescribing or dosing is reduced using the CalcVAN app in clinical practice.

## Declaration of Conflicting Interest

The authors declared no potential conflicts of interest with respect to the research, authorship, and/or publication of this article.

## Source of funding

This work was supported in part by 10.13039/501100003593National Council for Scientific and Technological Development (CNPq) and Federal Rural University of Rio de Janeiro, Brazil through scholarships. The funders had no role in study design, development of the mobile app, writing of the report or decision to publish.
